# Effects of functional and analytical strength training on upper-extremity activity after stroke: a randomized controlled trial

**DOI:** 10.1590/bjpt-rbf.2014.0187

**Published:** 2016-09-22

**Authors:** Patrícia Graef, Stella M. Michaelsen, Maria L. R. Dadalt, Daiana A. M. S. Rodrigues, Franciele Pereira, Aline S. Pagnussat

**Affiliations:** 1 Programa de Pós-graduação em Ciências da Reabilitação, Universidade Federal de Ciências da Saúde de Porto Alegre (UFCSPA), Porto Alegre, RS, Brazil; 2 Escola da Saúde, Centro Universitário Ritter dos Reis (UNIRITTER), Porto Alegre, RS, Brazil; 3 Programa de Pós-graduação em Ciências da Saúde, UFCSPA, Porto Alegre, RS, Brazil; 4 Programa de Pós-graduação em Fisioterapia, Universidade do Estado de Santa Catarina (UDESC), Florianópolis, SC, Brazil; 5 Departamento de Fisioterapia, UFCSPA, Porto Alegre, RS, Brazil

**Keywords:** stroke, neurological rehabilitation, physical therapy, resistance training, upper-limb

## Abstract

**Objective:**

To investigate the effects of functional strengthening (using functional movements) and analytical strengthening (using repetitive movements) on level of activity and muscular strength gain in patients with chronic hemiparesis after stroke.

**Method:**

A randomized, assessor-blinded trial was conducted in a therapist-supervised home rehabilitation program. Twenty-seven patients with chronic stroke were randomly allocated one of two groups: functional strengthening (FS) (n=13) and analytical strengthening (AS) (n=14). Each group received a five-week muscle strengthening protocol (30 minutes per day, three times per week) including functional movements or analytical movements, respectively. Pre-, post-, and ten-month follow-up outcomes included the Upper-Extremity Performance Test (primary outcome), Shoulder and Grip Strength, Active Shoulder Range of Motion (ROM), the Fugl-Meyer Assessment, and the Modified Ashworth Scale (MAS) (secondary outcomes).

**Results:**

There was significant improvement in the Upper-Extremity Performance Test for the combined unilateral and bilateral task scores in the FS Group (mean difference 2.4; 95% CI=0.14 to 4.6) in the 10-month follow-up. No significant difference was observed between groups in the other outcomes (p>0.05).

**Conclusion:**

A five-week home-based functional muscle strengthening induced positive results for the upper-extremity level of activity of patients with moderate impairment after chronic stroke.

## Bullet points

• Immediately after functional strengthening, patients with chronic hemiparesis and moderate motor deficits show improvement in activity levels for the paretic upper limb, and this improvement is maintained in follow-up.
• No between-group differences were found between groups in Shoulder and Grip Strength or in active Shoulder ROM.
• Both muscle strengthening protocols (functional or analytical) can be applied in patients with chronic hemiparesis and moderate motor deficit, without side effects and with improved functional strengthening at follow-up.

## Introduction

Stroke often causes significant disability^1^. Upper-extremity (UE) function is one of the most persistent and significant stroke-related physical impairments^2^. UE weakness occurs frequently after stroke and may compromise activities of daily living and limit function in individuals with hemiparesis^3^ ^,^ ^4^.

Muscular strength deficits are not always the main outcome following neurological damage. However, it can accompany other motor problems and is recognized as a limiting factor for rehabilitation^5^ ^-^ ^11^. Muscular weakness is associated with loss of motor units, deficient motor unit recruitment, inadequate firing frequencies to sustain muscle contraction, and muscle fiber atrophy^12^ ^,^ ^13^. Muscular weakness correlates with functional motor performance in patients with hemiparesis^14^. Therefore, strengthening interventions can increase muscle strength, promote functional improvement, and potentially change quality of life without negative side effects (such as the increase in hypertonia and pain)^4^ ^,^ ^15^.

Several studies have reported that activity-dependent brain plasticity is proportional to the complexity of motor learning and correlates with functional recovery after stroke^16^ ^-^ ^18^. In patients with chronic stroke, the isolated benefits of strength training performed with non-functional movements (analytical strength)^4^ and skill training^19^ ^,^ ^20^ have been described^21^ ^-^ ^25^. However, previous studies have not examined both protocols (functional training and muscle strength) to investigate UE activity levels after stroke. In a case study, Patten et al.^11^ demonstrated that the combination of functional training and dynamic high-intensity resistance training might be able to induce positive clinical effects. This training may also be able to promote improvements in UE function and enhance the quality of movement without deleterious effects including exacerbation of spasticity and musculoskeletal damage. Donaldson et al.^26^ performed a randomized controlled trial with patients in the post-stroke subacute phase (3 months after the injury) comparing conventional physical therapy with functional strengthening. This study showed no difference between groups but reported a trend for greater improvements in the functional strengthening group. Although it has been demonstrated that shoulder flexion and handgrip strength is strongly related to UE function^27^, no study has directly compared the effect of analytic versus functional strengthening on the recovery of functionality and muscular strength gain in patients with chronic stroke. This seems particularly important for chronic patients, taking into account that two to six months after stroke a substantial remodeling of motor units and muscles may occur and compromise long-term functional abilities^28^. Even though it is very well established that post-stroke therapy should include task-oriented training^22^ ^,^ ^29^ ^,^ ^30^ and strengthening interventions^8^, hemiparetic weakness and its rehabilitation remain poorly understood, especially in the UE.

Therefore, the aim of this study was to determine whether a five-week home-based program of functional strengthening was more effective than analytical strengthening for improving UE activity levels in subjects with chronic hemiparesis.

## Method

### Trial design

This prospective, single-blinded randomized clinical trial was registered and allocated by the Brazilian Clinical Trials Registry (identifier number RBR 22Y7P9). It included concealed randomization and blinded assessments according to the CONSORT guidelines for randomized clinical trials^31^ ^,^ ^32^. Ethical approval was given by the Ethics Committee of Universidade Federal de Ciências da Saúde de Porto Alegre (UFCSPA), Porto Alegre, RS, Brazil (protocol number 11–822). Written informed consent was obtained from all participants.

### Participants and randomization

Participants were recruited from Mãe de Deus Hospital and Grupo Hospitalar Conceição in Porto Alegre, Brazil, by reviewing medical records of patients admitted in the previous 5 years. The inclusion criteria consisted of: (1) six months to five years since the onset of a unilateral stroke; (2) ability to understand simple instructions (Mini-Mental State Examination with a minimum score of 20)^33^; (3) no pain, contractures, or severe weakness in the shoulder flexion muscles (<3 in Manual Muscle Testing); and (4) no UE rehabilitation while participating in this trial. The exclusion criteria included: (1) other neurological, neuromuscular, or orthopedic diseases; (2) severe comorbidity diseases; or (3) severe increase in UE muscular tone (>3 points according to the modified Ashworth scale)^34^.

Patients were matched based on muscle strength since it is an important factor in determining outcomes^27^. Participants were stratified based on maximum isometric force of shoulder flexion measured with a load cell (Miotec Equipamentos Biomédicos Ltda., Porto Alegre, RS, Brazil). They were instructed to perform three attempts and their best attempt was used for the following classification: weak (<2 kg), moderate (>2 kg and <4 kg), and strong (>4 kg). This stratification was performed in order to ensure sample homogeneity. Random assignment was computer generated by a person who was not involved in patient selection. The allocation schedule was generated and concealed in sequentially numbered, sealed, opaque envelopes. Participants were randomly assigned to two intervention groups: functional strengthening (FS) or analytical strengthening (AS) (n=14 each) one week before the start of the intervention.

### Intervention

All participants have received a 30-minute therapist-supervised home rehabilitation program three times per week for five weeks (total of 15 sessions)^28^. Each session began with a period of stretching and passive range of motion performed by the physical therapist. For both groups, the exercises were undertaken with participants seated in a chair, which allowed for a posture in which the knees and hips were maintained at 90°. All patients had their trunk restrained in order to avoid upper, anterior, lateral, or rotational trunk displacements during the strength training^35^ ^,^ ^36^.

The load was set according to the ability to generate maximal force during shoulder flexion, as mentioned before. The weight used during exercises remained at 60% of maximum strength measured during the baseline evaluation^37^. Both strengthening protocols began with range of motion at 60° of shoulder flexion and progressed to 90° by the eighth session. Participants were reassessed at the completion of the intervention phase (outcome) and 10 months post-intervention (follow-up). Every effort was made to invite subjects for assessment at outcome and follow-up even if they had withdrawn from therapy (intention-to-treat principle).

FS Group: Participants assigned to the FS group performed reaching-to-grasp movements against resistance. This intervention was designed to incorporate the repetitive nature of functional tasks and encouraged different grasping configurations by using objects that varied in size and shape. The previously established weight for each participant was placed within the object and remained throughout the intervention. Movements involving shoulder abduction, flexion, and adduction were performed to reach and grasp objects (plastic containers) at different heights. Participants were instructed to extend and flex their fingers to pick up a pot that was on a lower surface, place it on a higher surface, drop it, then pick up another pot with different diameter and place it on a lower surface, and so forth. During the first eight sessions of the protocol, a shoulder range of motion of 60 degrees was used, and in the remaining seven sessions, the shoulder range of motion was increased to 90 degrees. This range of motion was selected in order to avoid secondary pain as result of possible scapular dyskinesia. Even though the size of the pots could vary, the weight was kept constant.

AS Group: Participants assigned to the AS group performed UE strengthening using repetitive movements without a functional goal. Repetitive exercises were performed with a dumbbell (which was placed in the patient’s hand by the therapist) in shoulder abduction, flexion, and adduction with range of motion similar to the FS group (60 and 90 degrees progressively).

Both groups were instructed to perform at the same level of intensity and the same number of sessions. The final range of motion (60 or 90 degrees) was visually controlled by the therapists. The programs consisted of three sets of 12 repetitions (four repetitions for each movement direction – abduction, flexion, and adduction)^28^, with a three-minute rest period between sets. The physical therapists received training by the same instructor and used similar verbal cues for patients in both groups. Both protocols were conducted as home-based therapy. Blood pressure and cardiac frequency measurements were obtained before and after the interventions.

### Outcomes

#### Primary outcome measures

Measurements were performed at baseline, immediately after treatment (outcome), and 10 months after randomization. All measures were taken at the patient’s home by a research assistant who was blinded to group allocation. The primary clinical outcome was activity level (improvements in unilateral and bilateral UE performance) and was assessed by The Upper-Extremity Performance Test (Test d’Évaluation des Membres Supérieurs des Personnes Âgées - TEMPA)^38^. This test is used to evaluate the UE activity levels during the performance of functional activities. The TEMPA scale is composed of eight standardized tasks and four bilateral and unilateral tasks, which represents activities of daily living. Each task was evaluated with three criteria: speed of execution, functional rating, and task analysis. In this study, the criterion speed of execution was not used because we consider that great speed of execution does not necessarily correspond to a higher quality of movement in stroke patients. The functional rating refers to the participant’s autonomy in each task measured on a four-level scale: (0) successfully completed without hesitation or difficulty; (-1) completed, but with some difficulty; (-2) partially executed or some steps were performed with significant difficulty; and (-3) not completed, even if any degree of assistance was offered. The analyses of the performed tasks quantified the abilities and the difficulties according to five dimensions related to UE sensory motor skills: strength, range of motion, precision of gross movements, grip, and precision of fine movements, which are also scored from 0 to (-3). The total score was determined by adding the scores obtained for both the unilateral and bilateral tasks. Individual analysis of the unilateral and bilateral scores could allow the evaluation of real functional improvement in the affected UE. Scores ranged from 0 to -150, with higher scores representing better performance. Adequate reliability has been reported for adults with hemiparesis^38^.

#### Secondary outcome measures

Secondary outcome measures included shoulder and grip strength, active shoulder range of motion (ROM), motor recovery of the UE, and muscle tone. Three grip strength measures were recorded in a standardized position and instruction using the Jamar® dynamometer (Lafayette Instrument, Lafayette, IN, USA). Three shoulder strength measures were also recorded in a standardized position and instruction with a load cell (Miotec Equipamentos Biomédicos Ltda., Porto Alegre, RS, Brazil). The highest score was used for the analysis. Standard goniometry was used to measure active shoulder flexion ROM. The improvement in motor impairment of the UE was evaluated with the UE section of the Fugl-Meyer (UE-FM) assessment scale^39^. UE-FM is a motor impairment test that includes four motor sub-items relevant to the involved UE: (1) shoulder/elbow/forearm, (2) wrist, (3) hand, and (4) speed coordination. Each item was rated on a three-point scale (0=cannot perform; 1=partially performed; 2=fully performed) for a 66-point maximum. The modified Ashworth scale^40^ was used to evaluate muscle tone.

### Sample size

The primary clinical outcome (TEMPA scale) was chosen for the sample size calculation. Based on our previous data^39^, we calculated the sample size to detect a difference of 7.0 (estimated SD=10) points in the combined unilateral and bilateral task scores, with a power of 80% for a two-tailed t-test with significance level set at 0.05 (32 patients per group).

### Statistical analysis

Results are presented as median (min-max) or mean and standard deviation (SD). Data normality was tested using the Shapiro-Wilk test, and the homogeneity of variance was tested by Levene’s test. Only grip force and active shoulder ROM showed normality. To determine differences in those evaluations, a two-way analysis of variance (ANOVA) test was used (group and time as factors). For non-parametric data, the Mann-Whitney U-test was used to compare the scores between groups. In the case of missing follow-up values, the last-observation-carried-forward (LOCF) method was used. This method inputs the outcome measures as follow-up determination. SPSS® 16.0 (Statistical Package for the Social Sciences, Inc., Chicago, IL, USA) was used for data analysis. Significance was set at p<0.05.

## Results

**Figure 1:**
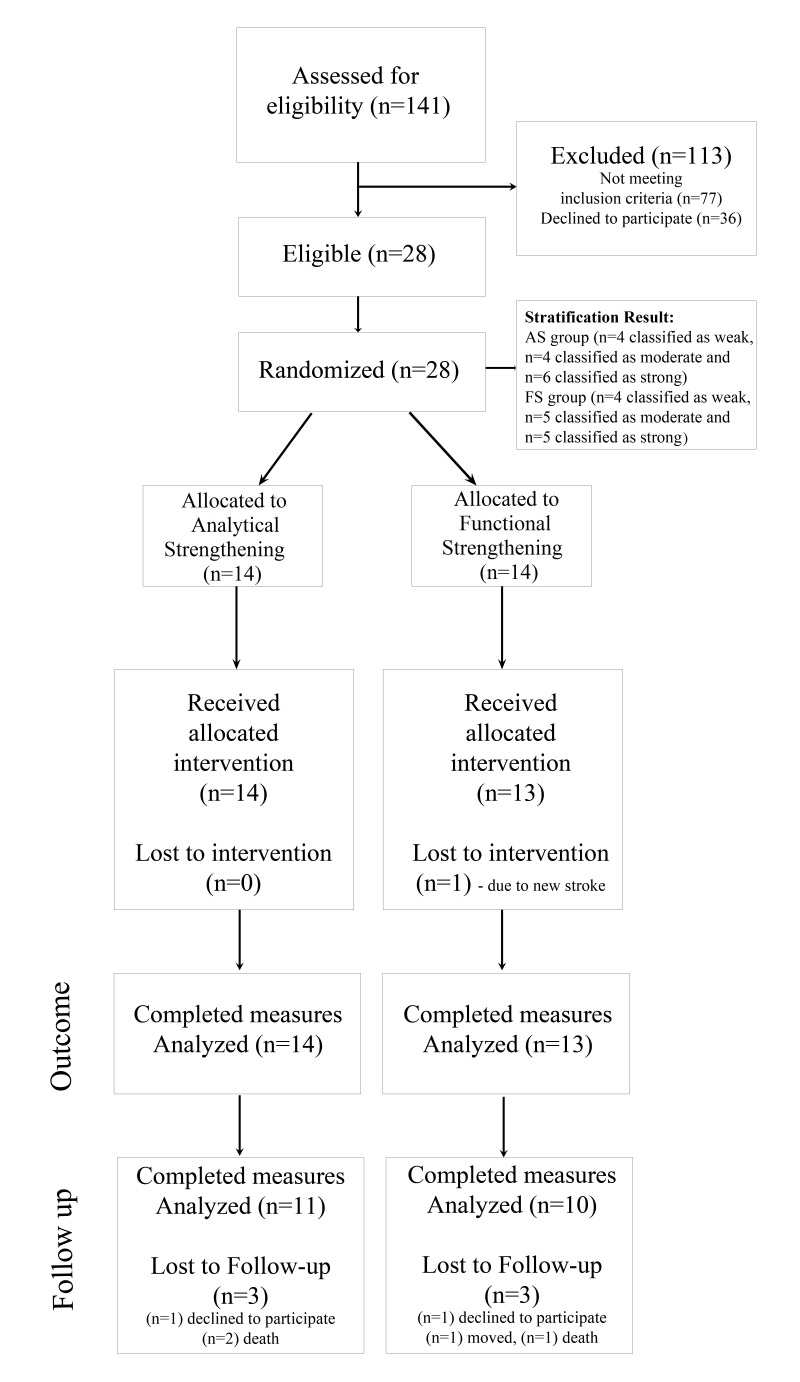
Subject Recruitment and Attrition Flowchart. AS: Analytical Strengthening; FS: Functional Strengthening.

A total of 141 patients were screened, of whom 28 (20%) met the study criteria. One subject from the FS group dropped out before the baseline measure due to a second stroke. Consequently, the 27 participants were randomized as follows: 13 to FS and 14 to AS. After completion of the intervention, all patients performed the outcome measures. In the 10-month follow-up, six participants could not be reassessed due to death (3), unwillingness to participate (2), or relocation (1) (Figure 1).

The biographical characteristics of the 27 patients are shown in Table 1. In summary, the mean age of the total study sample was 67.8±12.3 years, and 16/27 were women. Mean time post-onset was 2.5±1.4 years. In 70% of the sample, stroke occurred in the left hemisphere. This sample was classified with mild/moderate impairment according to the Fugl-Meyer Assessment (UE-FM scores between 28 and 50)^41^. Attribute data were similar between groups. Groups were similar at baseline. No important adverse events or side effects occurred in the intervention groups.

**Table 1:** Characteristics of the participants.

	FS (n=13)	AS (n=14)
Gender, n (%)		
Male	6 (46.0%)	5 (36.0%)
Female	7 (54.0%)	9 (64.0%)
Paretic side, n (%)		
Right	8 (62.0%)	11 (79.0%)
Left	5 (38.0%)	3 (21.0%)
Age (years - mean±SD)	72±12	63±11
Time since onset (years - mean±SD)	2.0±1.4	2.8±1.4

FS: Functional Strength Training; AS: Analytical Strength Training.

Table 2 shows the mean and standard deviation of the primary and secondary outcomes. Table 3 shows the between-group analysis for all comparisons.

**Table 2:** Means (SD) and Median (min-max) at pre-pest, post-test, and follow-up for patients with chronic hemiparesis after stroke who received functional strengthening or analytical strengthening.

	**Intervention**					
**Outcome**	**Pre-test**		**Post-test**		**Follow-up**	
	**FS**	**AS**	**FS**	**AS**	**FS**	**AS**
TEMPA Functional Rating						
Unilateral Tasks (0 to -12)	-3.84 (3.38)	-5.78 (4.64)	-3.23 (3.65)	-5.28 (4.92)	-2.61 (3.42)	-5.0 (4.97)
Bilateral Tasks (0 to -12)	-0.76 (1.36)	-1.92 (2.86)	-0.30 (0.63)	-1.92 (2.84)	-0.76 (1.23)	-1.92 (2.89)
TEMPA Task Analysis						
Unilateral Tasks (0 to -12)	-13 (13.34)	-20.78 (19.32)	-10.69 (13.49)	-19.57 (20.34)	-8.84 (13.17)	-18.64 (20.41)
Bilateral Tasks (0 to -12)	-6.69 (7.02)	-10.35 (10.22)	-4.69 (7.40)	-8.35 (9.72)	-5.07 (7.55)	-8.5 (10.78)
TEMPA – Unilateral Total Score (0 to -60)	-17.15 (16.69)	-25.64 (24.74)	-13.92 (17.10)	-24.50 (25.55)	-11.46 (16.52)	-22.85 (25.93)
TEMPA – Bilateral Total Score (0 to -66)	-7.46 (8.34)	-12.28 (12.89)	-5.0 (8.0)	-10.28 (12.47)	-5.84 (8.73)	-10.42 (13.55)
TEMPA – Unilateral and Bilateral Tasks Scores Combined (0 to -186)	-25.07 (23.90)	-38.85 (35.77)	-18.92 (24.55)	-35.14 (35.92)	-17.46 (24.66)	-33.78 (35.50)
Shoulder Flexors (kg)	3.90 (2.34)	3.10 (1.79)	7.85 (4.98)	8.93 (7.17)	7.93 (5.22)	6.83 (4.86)
Hand Grip (pounds)	31.38 (12.41)	23.85 (13.52)	38.38 (13.90)	30.0 (15.28)	39.07 (17.41)	27.0 (17.21)
Active shoulder ROM (degrees)	99.61 (39.39)	86.42 (32.36)	121.23 (37.33)	102.85 (32.32)	128.07 (33.82)	107.5 (31.66)
FM (0-66)	49.30 (11.81)	43.92 (12.25)	58.07 (9.35)	51.42 (12.68)	59.53 (6.72)	52.42 (12.01)
Muscle tone (Modified Ashworth Scale 0 - 4)	1 (0-2)	1.5 (0-3)	1 (0-2)	1 (0-3)	1(0-2)	1(0-3)

Data are expressed as mean with standard deviation (SD) and median (min-max) for muscle tone.

**Table 3:** Between-group differences at post-test and follow-up after randomization for patients with chronic hemiparesis after stroke.

**Outcome**		**Difference between interventions Adjusted Mean Difference (95% CI)**						
		**Pre-test to Post-test** **(95%CI), p**				**Pre-test to Follow-up** **(95%CI), p**		
		**FSxAS**		**p**		**FSxAS**		**p**
TEMPA Functional Rating								
Unilateral Tasks (0 to -12)	0.11 (-0.7 to 0.47)				0.68		0.44 (-1.32 to 0.43)	0.31
Bilateral Tasks (0 to -12)	0.5 (-1.13 to 0.13)				0.12		0 (-0.71 to 0.71)	0.99
TEMPA Task Analysis								
Unilateral Tasks (0 to -12)	1.4 (-2.98 to 0.18)		0.05				2.32 (-5.32 to 0.69)	0.14
Bilateral Tasks (0 to -12)	0.58 (-3.12 to 1.95)		0.64				0.24 (-3.42 to 3.9)	0.89
TEMPA – Unilateral Total Score (0 to -60)	1.86 (-3.41 to -0.31)		0.02*				2.9 (-6.62 to 0.81)	0.13
TEMPA – Bilateral Total Score (0 to -66)	0.46 (-3.48 to 2.55)		0.75				0.24 (-3.92 to 0.44)	0.90
TEMPA – Unilateral and Bilateral Tasks Scores Combined (0 to -186)	2.44 (-4.88 to -0.03)		0.05*				2.4 (0.14 to 4.6)	0.03*
Shoulder Flexors (kg)	1.9 (-2.48 to 6.28)		0.37				0.3 (-3.27 to 2.67)	0.83
Hand Grip (pounds)	1 (-7.48 to 5.48)		0.75				5 (-15.25 to 5.25)	0.34
Active shoulder ROM (degrees)	5.18 (-19.97 to 9.59)		0.48				7.39 (-24.97 to 10.19)	0.41
FM (0-66)	1.27 (-4.31 to 1.77)		0.40				1.73 (-5.77 to 2.31)	0.39
Muscle tone (Modified Ashworth Scale 0 - 4)	0.1 (-0.35 to 0.57)		0.67				0 (-0.43 to 0.43)	0.99

* Significant difference (p<0.05).

### Primary outcome measure

The TEMPA scores significantly improved in both groups throughout the intervention period (outcome measures) and in the follow-up. A statistically significant difference was observed between the FS group and the AS group for the unilateral task analysis (mean difference of 1.86; 95% CI=-3.41 to -0.31) immediately after treatment and the combined unilateral and bilateral task scores (mean difference 2.4; 95% CI=0.14 to 4.6) in the 10-month follow-up (Tables 2 and 3).

### Secondary outcome measures

No statistically significant differences were observed for handgrip strength and for shoulder flexion strength between groups immediately after treatment or in the 10-month follow-up. For active shoulder ROM and for the motor assessment (UE-FM), no difference between group effects was observed.

Muscle tone evaluation (Ashworth scale) demonstrated no difference between groups immediately after treatment or in the 10-month follow-up (Table 3).

## Discussion

This study was performed to determine the effect of functional and analytical strength training on UE activity levels in patients with chronic stroke. Observations made in this randomized trial partially confirmed our preliminary hypothesis. Functional strength training was able to induce greater improvements in the combined unilateral and bilateral activity of the paretic UE, as evaluated by means of the TEMPA scale immediately after the treatment and in the 10-month follow-up.

Although morphological and physiological changes in motor units have been observed in patients with chronic stroke^12^ ^,^ ^13^, earlier studies reported the possibility of improving muscle strength in the UE^4^ ^,^ ^22^ ^,^ ^28^ ^,^ ^42^ with rehabilitation that included muscle strength training. Our results corroborate and add to the previous findings. We have demonstrated that resistance weight training leads to significant gains in handgrip and shoulder flexion strength for both groups. These muscles were selected since they are major predictors for paretic UE function after stroke^27^. The lack of significant differences between groups for shoulder flexion and handgrip strength was partially expected since all patients maintained essentially the same training intensity, volume, and frequency.

Another relevant factor for the improvement in activities of daily living is the active shoulder ROM. The loss of elbow-shoulder coordination and the decreased active ROM partly explain differences in movement patterns between stroke patients and healthy subjects^43^. In addition, it may be caused by weakness or altered recruitment of muscles directly involved in the synergistic patterns of movement^44^ ^,^ ^45^. It has also been suggested that a decreased ability to regulate stretch-reflex thresholds and to coordinate changes in thresholds for a group of muscles may also cause restrictions in ROM and affect postural stability^43^ ^,^ ^46^. We have demonstrated an increased active shoulder ROM with concurrent enhancement of strength and UE motor function. The UE-FM scale is the predominant tool to evaluate motor impairment after stroke and it assesses the presence of synergistic versus isolated patterns of movement^45^. Our findings showed no difference between groups regarding motor control enhancement. The improvement observed for both groups might be considered clinically important, as demonstrated by a 4.25 point increase in the scale^41^.

Muscle weakness is a significant motor impairment that mainly hinders voluntary movements^14^, and UE strengthening has been extensively shown to positively influence motor control^8^ ^,^ ^14^ ^,^ ^18^ ^,^ ^40^. The impairment of UE movements and strength after stroke may be viewed as a deficit in motor execution and a deficit in higher-order processes (motor planning and motor learning). This can lead to poorly formed sensorimotor associations or internal representations^47^. Muscle strength may be one of the main determinants for the improvement of motor control in patients with chronic stroke since no difference was observed between groups.

Current neurorehabilitation approaches advocate the use of functional tasks compared to performing systematic strength training with isolated exercises^29^ ^,^ ^30^. Movement learning depends on the protein synthesis, synaptogenesis, and map changes in the primary motor cortex (M1)^16^ ^,^ ^48^. There are several studies that suggest that activity-dependent brain plasticity is proportional to the complexity of motor learning and that strength training alone fails to change cortical M1 somatotopy^49^ ^-^ ^52^. Corti et al.^35^ proposed that power training, when performed before a functional task, could significantly reduce trunk displacement during UE movements and that this reduced compensation would be accompanied by the reappearance of normal movement patterns. We demonstrated that functional strengthening was greater when we compared the results of the analytical training group in the analysis of the total score of unilateral and bilateral UE activities. This result indicates a general improvement in the functional parameters and qualitative analysis evaluated by the TEMPA.

One important point is that both strengthening protocols used in this trial induced no increase in muscle tone, agreeing with recent studies that have demonstrated the benefits of muscle strengthening without detrimental effects to patients after stroke, such as pain or exacerbation of spasticity^11^ ^,^ ^26^. Therefore, resistance weight training could improve UE muscle strength in the paretic limb of patients with chronic stroke, which carry over to improvements in motor control.

This study had some limitations. One limitation was the relatively small sample size. This sample did not reach the calculated sample size due to the specific inclusion and exclusion criteria. After intervention, we analyzed more than 90% of the sample, but the sample loss at follow-up was more than 20% of sample loss because of death, relocation, or unwillingness to participate. Our results are extended specifically to patients with chronic stroke and moderate paresis. Another limitation is the absence of a control group that received neither functional strength training nor analytical strength training. For this reason, our findings cannot be generalized to the broader community based on this study alone.

In the present study, we reported positive results for muscle strength training during UE rehabilitation in patients with chronic stroke. Immediately after functional strengthening, patients improved their activity level for the paretic UE and this improvement was maintained in the follow-up. Therefore, a 5-week home-based functional muscle strengthening induced positive results for the UE activity levels of patients with chronic hemiparesis and moderate motor deficits. These findings have important implications for the rehabilitation of patients with chronic stroke and moderate hemiparesis.
